# *cis*-Dimethyl­bis(triphenyl­arsine)platinum(II)

**DOI:** 10.1107/S1600536809055470

**Published:** 2010-01-16

**Authors:** Anwar Abo-Amer

**Affiliations:** a Chemistry Department, Al al-Bayt University, Al-Mafraq, Jordan

## Abstract

In the title compound, [Pt(CH_3_)_2_(C_18_H_15_As)_2_], the Pt^II^ atom adopts a distorted *cis*-PtAs_2_C_2_ square-planar coordination geometry. In the crystal, mol­ecules inter­act *via* aromatic π–π stacking inter­actions [centroid–centroid separation = 3.6741 (18) Å].

## Related literature

For the structures of related complexes, see: Anderson *et al.* (1982 ▶); Al-Fawaz *et al.* (2004 ▶); Fun *et al.* (2006 ▶). For further synthetic details, see: Puddephatt *et al.* (1998 ▶).
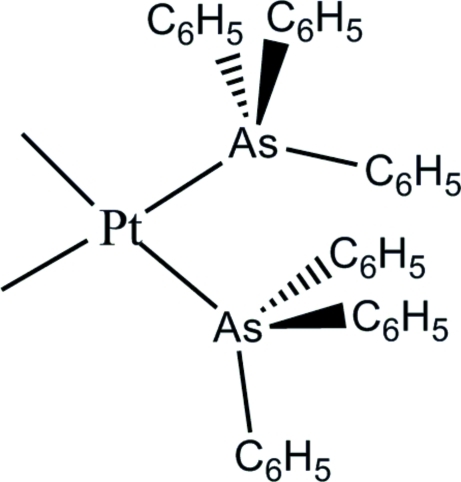


## Experimental

### 

#### Crystal data

[Pt(CH_3_)_2_(C_18_H_15_As)_2_]
*M* *_r_* = 837.60
Triclinic, 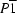

*a* = 10.1033 (9) Å
*b* = 10.3937 (8) Å
*c* = 17.2452 (13) Å
α = 91.106 (2)°
β = 99.588 (2)°
γ = 115.227 (2)°
*V* = 1607.0 (2) Å^3^
*Z* = 2
Mo *K*α radiation
μ = 6.43 mm^−1^
*T* = 150 K
0.22 × 0.20 × 0.19 mm

#### Data collection

Bruker APEXII CCD diffractometer
Absorption correction: multi-scan (*SADABS*; Bruker, 2001 ▶) *T*_min_ = 0.332, *T*_max_ = 0.374
19166 measured reflections
9358 independent reflections
8543 reflections with *I* > 2σ(*I*)
*R*_int_ = 0.019

#### Refinement

*R*[*F* ^2^ > 2σ(*F* ^2^)] = 0.020
*wR*(*F* ^2^) = 0.077
*S* = 0.64
9358 reflections
372 parameters
H-atom parameters constrained
Δρ_max_ = 0.56 e Å^−3^
Δρ_min_ = −1.07 e Å^−3^

### 

Data collection: *APEX2* (Bruker, 2001 ▶); cell refinement: *SAINT* (Bruker, 2001 ▶); data reduction: *SAINT*; program(s) used to solve structure: *SHELXS97* (Sheldrick, 2008 ▶); program(s) used to refine structure: *SHELXL97* (Sheldrick, 2008 ▶); molecular graphics: *SHELXTL* (Sheldrick, 2008 ▶); software used to prepare material for publication: *SHELXTL*.

## Supplementary Material

Crystal structure: contains datablocks I, global. DOI: 10.1107/S1600536809055470/hb5294sup1.cif

Structure factors: contains datablocks I. DOI: 10.1107/S1600536809055470/hb5294Isup2.hkl

Additional supplementary materials:  crystallographic information; 3D view; checkCIF report

## Figures and Tables

**Table 1:** Selected bond lengths (Å)

Pt1—C20	2.060 (3)
Pt1—C19	2.083 (3)
Pt1—As1	2.3960 (3)
Pt1—As2	2.4086 (3)

**Fig. 1.**
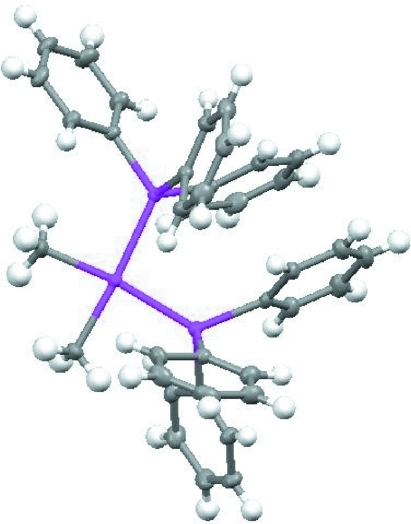
The molecular structure of (I) showing 50% displacement ellipsoids (H atoms omitted for clarity).

## supplementary crystallographic information

### Comment

*cis*- and *trans*-Dimethylebis(triphenylarsine)platinum(II) are useful to prepare other platinum complexes. The structures of the *cis*-isomer have been not reported. During the course of our studies on platinium sulfide compounds *cis*-[Pt_2_Me_4_(µ-SMe_2_)_2_] with Lewis bases, a colorless crystals of the title compound, (I), was obtained. As shown in Fig.1, the platinum centre is four-coordinated and adopts a nearly square planar geometry. The Pt—As bond lengths (2.3960 (2) and 2.4086 (2) Å) and Pt—C bond lengths (2.060 (3) and 2.083 (3) Å), as well as the bond angles around the Pt atom are similar to those in the above-mentioned structures.

### Experimental

The title complex was prepared by the addition of an excess of AsPh_3_(0.2133 mmol) to an anhydrous diethyl ether solution (20 ml) of *cis*-[PtMe_2_(SMe_2_)_2_]_2_ (0.17413 mmol) (Puddephatt *et al.* 1998) at 298 K. The desired product precipitates spontaneously in near quantitative yield (78.9% yield) and was recrystallized from dichloromethane at 298 K to yield colourless prisms of (I).

### Refinement

Thelargest peak in the final difference electron density synthesis was 0.559 e^-^/Å^3^ and the largest hole was -1.070 e^-^/Å^3^ with an RMS deviation of 0.104 e^-^/Å^3^.

### Crystal data

**Table:**

[Pt(CH_3_)_2_(C_18_H_15_As)_2_]	*Z* = 2
*M**_r_* = 837.60	*F*(000) = 816
Triclinic, *P*1	*D*_x_ = 1.731 Mg m^−^^3^
Hall symbol: -P 1	Mo *K*α radiation, λ = 0.71073 Å
*a* = 10.1033 (9) Å	Cell parameters from 9885 reflections
*b* = 10.3937 (8) Å	θ = 2.3–30.2°
*c* = 17.2452 (13) Å	µ = 6.43 mm^−^^1^
α = 91.106 (2)°	*T* = 150 K
β = 99.588 (2)°	Prism, colourless
γ = 115.227 (2)°	0.22 × 0.20 × 0.19 mm
*V* = 1607.0 (2) Å^3^	

### Data collection

**Table:**

Bruker APEXII CCD diffractometer	9358 independent reflections
Radiation source: fine-focus sealed tube	8543 reflections with *I* > 2σ(*I*)
graphite	*R*_int_ = 0.019
φ and ω scans	θ_max_ = 30.2°, θ_min_ = 1.2°
Absorption correction: multi-scan (*SADABS*; Bruker, 2001)	*h* = −14→13
*T*_min_ = 0.332, *T*_max_ = 0.374	*k* = −14→14
19166 measured reflections	*l* = −24→24

### Refinement

**Table:**

Refinement on *F*^2^	Primary atom site location: structure-invariant direct methods
Least-squares matrix: full	Secondary atom site location: difference Fourier map
*R*[*F*^2^ > 2σ(*F*^2^)] = 0.020	Hydrogen site location: inferred from neighbouring sites
*wR*(*F*^2^) = 0.077	H-atom parameters constrained
*S* = 0.64	*w* = 1/[σ^2^(*F*_o_^2^) + (0.1*P*)^2^] where *P* = (*F*_o_^2^ + 2*F*_c_^2^)/3
9358 reflections	(Δ/σ)_max_ = 0.066
372 parameters	Δρ_max_ = 0.56 e Å^−^^3^
0 restraints	Δρ_min_ = −1.07 e Å^−^^3^

### Special details

**Table:**

Geometry. All e.s.d.'s (except the e.s.d. in the dihedral angle between two l.s. planes) are estimated using the full covariance matrix. The cell e.s.d.'s are taken into account individually in the estimation of e.s.d.'s in distances, angles and torsion angles; correlations between e.s.d.'s in cell parameters are only used when they are defined by crystal symmetry. An approximate (isotropic) treatment of cell e.s.d.'s is used for estimating e.s.d.'s involving l.s. planes.
Refinement. Refinement of *F*^2^ against ALL reflections. The weighted *R*-factor *wR* and goodness of fit *S* are based on *F*^2^, conventional *R*-factors *R* are based on *F*, with *F* set to zero for negative *F*^2^. The threshold expression of *F*^2^ > σ(*F*^2^) is used only for calculating *R*-factors(gt) *etc*. and is not relevant to the choice of reflections for refinement. *R*-factors based on *F*^2^ are statistically about twice as large as those based on *F*, and *R*- factors based on ALL data will be even larger.

### Fractional atomic coordinates and isotropic or equivalent isotropic displacement parameters (Å^2^)

**Table:**

	*x*	*y*	*z*	*U*_iso_*/*U*_eq_	
Pt1	1.029195 (9)	0.072490 (9)	0.271372 (5)	0.01642 (4)	
As1	1.04466 (3)	0.25078 (3)	0.367248 (15)	0.01542 (6)	
As2	0.85760 (3)	0.10672 (3)	0.168572 (15)	0.01719 (6)	
C1	0.8543 (3)	0.2235 (3)	0.39079 (15)	0.0188 (4)	
C2	0.8076 (3)	0.3307 (3)	0.39670 (16)	0.0236 (5)	
H2	0.8707	0.4259	0.3885	0.028*	
C3	0.6679 (3)	0.2982 (4)	0.41463 (17)	0.0311 (6)	
H3	0.6352	0.3710	0.4180	0.037*	
C4	0.5768 (3)	0.1597 (4)	0.42758 (19)	0.0357 (7)	
H4	0.4829	0.1382	0.4412	0.043*	
C5	0.6223 (3)	0.0536 (4)	0.42067 (19)	0.0336 (7)	
H5	0.5586	−0.0414	0.4288	0.040*	
C6	0.7605 (3)	0.0839 (3)	0.40192 (17)	0.0248 (5)	
H6	0.7908	0.0098	0.3967	0.030*	
C7	1.1385 (2)	0.4450 (3)	0.33885 (15)	0.0176 (4)	
C8	1.1859 (3)	0.4651 (3)	0.26647 (16)	0.0241 (5)	
H8	1.1696	0.3849	0.2322	0.029*	
C9	1.2570 (3)	0.6023 (3)	0.2447 (2)	0.0322 (6)	
H9	1.2887	0.6161	0.1954	0.039*	
C10	1.2818 (3)	0.7199 (3)	0.2954 (2)	0.0330 (7)	
H10	1.3291	0.8139	0.2803	0.040*	
C11	1.2374 (3)	0.6997 (3)	0.3677 (2)	0.0295 (6)	
H11	1.2559	0.7800	0.4026	0.035*	
C12	1.1659 (3)	0.5623 (3)	0.38953 (16)	0.0227 (5)	
H12	1.1358	0.5489	0.4393	0.027*	
C13	1.1613 (3)	0.2778 (3)	0.47356 (15)	0.0180 (4)	
C14	1.3155 (3)	0.3227 (3)	0.48190 (17)	0.0246 (5)	
H14	1.3604	0.3403	0.4366	0.029*	
C15	1.4027 (3)	0.3414 (3)	0.55628 (19)	0.0299 (6)	
H15	1.5072	0.3723	0.5617	0.036*	
C16	1.3385 (3)	0.3152 (3)	0.62213 (18)	0.0303 (6)	
H16	1.3988	0.3279	0.6728	0.036*	
C17	1.1872 (4)	0.2707 (4)	0.61499 (18)	0.0340 (7)	
H17	1.1434	0.2533	0.6607	0.041*	
C18	1.0979 (3)	0.2513 (3)	0.54014 (17)	0.0264 (5)	
H18	0.9934	0.2198	0.5352	0.032*	
C19	1.0329 (3)	−0.0808 (3)	0.19328 (19)	0.0294 (6)	
H19A	1.1365	−0.0600	0.1916	0.044*	
H19B	0.9808	−0.0790	0.1403	0.044*	
H19C	0.9833	−0.1757	0.2112	0.044*	
C20	1.1755 (3)	0.0233 (3)	0.34881 (18)	0.0297 (6)	
H20A	1.1554	−0.0765	0.3362	0.045*	
H20B	1.1627	0.0367	0.4030	0.045*	
H20C	1.2780	0.0862	0.3440	0.045*	
C21	0.7011 (3)	−0.0729 (3)	0.11442 (15)	0.0190 (4)	
C22	0.6602 (3)	−0.1928 (3)	0.15666 (18)	0.0266 (5)	
H22	0.7092	−0.1856	0.2097	0.032*	
C23	0.5468 (3)	−0.3232 (3)	0.1202 (2)	0.0320 (6)	
H23	0.5195	−0.4051	0.1486	0.038*	
C24	0.4745 (3)	−0.3344 (3)	0.0442 (2)	0.0314 (6)	
H24	0.3960	−0.4234	0.0206	0.038*	
C25	0.5153 (3)	−0.2165 (3)	0.00144 (18)	0.0286 (6)	
H25	0.4660	−0.2248	−0.0517	0.034*	
C26	0.6292 (3)	−0.0855 (3)	0.03672 (16)	0.0231 (5)	
H26	0.6577	−0.0046	0.0075	0.028*	
C27	0.9495 (3)	0.2048 (3)	0.08340 (16)	0.0208 (5)	
C28	1.0904 (3)	0.2177 (3)	0.07933 (18)	0.0256 (5)	
H28	1.1393	0.1803	0.1179	0.031*	
C29	1.1602 (3)	0.2855 (3)	0.0186 (2)	0.0338 (7)	
H29	1.2560	0.2925	0.0155	0.041*	
C30	1.0929 (4)	0.3417 (3)	−0.03621 (19)	0.0326 (7)	
H30	1.1421	0.3888	−0.0770	0.039*	
C31	0.9532 (4)	0.3303 (3)	−0.03262 (18)	0.0317 (6)	
H31	0.9059	0.3689	−0.0713	0.038*	
C32	0.8815 (3)	0.2632 (3)	0.02676 (17)	0.0260 (5)	
H32	0.7856	0.2566	0.0291	0.031*	
C33	0.7360 (3)	0.2037 (3)	0.18684 (15)	0.0196 (5)	
C34	0.7918 (3)	0.3515 (3)	0.18978 (17)	0.0236 (5)	
H34	0.8900	0.4068	0.1812	0.028*	
C35	0.7047 (3)	0.4193 (3)	0.20524 (18)	0.0297 (6)	
H35	0.7431	0.5203	0.2063	0.036*	
C36	0.5628 (4)	0.3401 (4)	0.2190 (2)	0.0328 (6)	
H36	0.5037	0.3866	0.2297	0.039*	
C37	0.5064 (3)	0.1928 (4)	0.21732 (19)	0.0322 (6)	
H37	0.4089	0.1383	0.2270	0.039*	
C38	0.5923 (3)	0.1251 (3)	0.20146 (17)	0.0261 (5)	
H38	0.5532	0.0241	0.2005	0.031*	

### Atomic displacement parameters (Å^2^)

**Table:**

	*U*^11^	*U*^22^	*U*^33^	*U*^12^	*U*^13^	*U*^23^
Pt1	0.01636 (6)	0.01714 (6)	0.01599 (6)	0.00868 (4)	0.00017 (4)	−0.00089 (4)
As1	0.01461 (10)	0.01646 (11)	0.01471 (12)	0.00679 (9)	0.00170 (9)	0.00018 (9)
As2	0.01673 (11)	0.01870 (12)	0.01557 (12)	0.00809 (9)	0.00068 (9)	0.00099 (9)
C1	0.0151 (9)	0.0246 (12)	0.0153 (11)	0.0081 (9)	0.0010 (8)	0.0000 (9)
C2	0.0226 (11)	0.0284 (13)	0.0212 (12)	0.0137 (10)	0.0009 (10)	−0.0017 (10)
C3	0.0269 (12)	0.0515 (18)	0.0212 (13)	0.0245 (13)	0.0012 (11)	−0.0013 (12)
C4	0.0186 (11)	0.064 (2)	0.0231 (14)	0.0167 (13)	0.0042 (10)	0.0064 (14)
C5	0.0202 (12)	0.0395 (17)	0.0292 (15)	0.0011 (11)	0.0062 (11)	0.0046 (13)
C6	0.0183 (10)	0.0248 (12)	0.0272 (14)	0.0060 (9)	0.0027 (10)	0.0022 (10)
C7	0.0157 (9)	0.0182 (11)	0.0183 (11)	0.0072 (8)	0.0023 (8)	0.0003 (9)
C8	0.0226 (11)	0.0270 (13)	0.0199 (12)	0.0084 (10)	0.0034 (10)	0.0001 (10)
C9	0.0307 (13)	0.0323 (15)	0.0314 (16)	0.0094 (12)	0.0110 (12)	0.0135 (12)
C10	0.0308 (14)	0.0239 (13)	0.0427 (18)	0.0112 (11)	0.0039 (13)	0.0125 (13)
C11	0.0284 (13)	0.0202 (12)	0.0389 (17)	0.0109 (10)	0.0031 (12)	0.0046 (11)
C12	0.0254 (11)	0.0216 (12)	0.0206 (12)	0.0104 (10)	0.0028 (10)	0.0006 (9)
C13	0.0173 (9)	0.0179 (10)	0.0159 (11)	0.0061 (8)	0.0007 (8)	0.0004 (8)
C14	0.0215 (11)	0.0284 (13)	0.0225 (13)	0.0103 (10)	0.0025 (10)	0.0026 (10)
C15	0.0206 (11)	0.0296 (14)	0.0328 (16)	0.0082 (10)	−0.0044 (11)	0.0038 (12)
C16	0.0316 (13)	0.0293 (14)	0.0228 (13)	0.0116 (11)	−0.0088 (11)	0.0032 (11)
C17	0.0377 (15)	0.0427 (17)	0.0181 (13)	0.0153 (14)	0.0019 (12)	0.0054 (12)
C18	0.0244 (12)	0.0352 (15)	0.0203 (13)	0.0127 (11)	0.0062 (10)	0.0057 (11)
C19	0.0348 (14)	0.0320 (15)	0.0272 (14)	0.0222 (12)	0.0009 (12)	−0.0050 (11)
C20	0.0353 (14)	0.0370 (15)	0.0254 (14)	0.0275 (13)	−0.0040 (11)	−0.0033 (12)
C21	0.0182 (10)	0.0194 (11)	0.0177 (11)	0.0074 (9)	0.0014 (9)	0.0016 (9)
C22	0.0256 (12)	0.0269 (13)	0.0258 (14)	0.0106 (10)	0.0024 (10)	0.0090 (11)
C23	0.0261 (12)	0.0236 (13)	0.0437 (18)	0.0082 (11)	0.0065 (12)	0.0107 (12)
C24	0.0195 (11)	0.0241 (13)	0.0434 (18)	0.0052 (10)	0.0000 (11)	−0.0032 (12)
C25	0.0224 (11)	0.0319 (14)	0.0252 (14)	0.0089 (11)	−0.0030 (10)	−0.0044 (11)
C26	0.0219 (11)	0.0240 (12)	0.0201 (12)	0.0083 (10)	−0.0001 (9)	0.0029 (10)
C27	0.0201 (10)	0.0197 (11)	0.0203 (12)	0.0065 (9)	0.0040 (9)	−0.0006 (9)
C28	0.0230 (11)	0.0260 (13)	0.0301 (14)	0.0115 (10)	0.0089 (10)	0.0005 (11)
C29	0.0327 (14)	0.0280 (14)	0.0405 (18)	0.0088 (12)	0.0192 (13)	0.0018 (12)
C30	0.0421 (16)	0.0236 (13)	0.0274 (15)	0.0064 (12)	0.0172 (13)	−0.0010 (11)
C31	0.0423 (16)	0.0275 (14)	0.0215 (14)	0.0108 (12)	0.0079 (12)	0.0051 (11)
C32	0.0277 (12)	0.0251 (13)	0.0225 (13)	0.0091 (10)	0.0042 (10)	0.0045 (10)
C33	0.0195 (10)	0.0270 (12)	0.0151 (11)	0.0131 (9)	0.0022 (9)	0.0039 (9)
C34	0.0265 (12)	0.0242 (12)	0.0227 (13)	0.0143 (10)	0.0024 (10)	0.0024 (10)
C35	0.0374 (15)	0.0319 (14)	0.0274 (14)	0.0220 (13)	0.0065 (12)	0.0031 (11)
C36	0.0394 (15)	0.0419 (17)	0.0301 (15)	0.0282 (14)	0.0104 (13)	0.0077 (13)
C37	0.0306 (13)	0.0448 (18)	0.0283 (15)	0.0203 (13)	0.0129 (12)	0.0112 (13)
C38	0.0261 (12)	0.0304 (14)	0.0247 (13)	0.0137 (11)	0.0079 (10)	0.0074 (11)

### Geometric parameters (Å, °)

**Table:**

Pt1—C20	2.060 (3)	C17—H17	0.9500
Pt1—C19	2.083 (3)	C18—H18	0.9500
Pt1—As1	2.3960 (3)	C19—H19A	0.9800
Pt1—As2	2.4086 (3)	C19—H19B	0.9800
As1—C1	1.935 (2)	C19—H19C	0.9800
As1—C7	1.943 (2)	C20—H20A	0.9800
As1—C13	1.951 (2)	C20—H20B	0.9800
As2—C27	1.940 (3)	C20—H20C	0.9800
As2—C21	1.945 (2)	C21—C26	1.388 (4)
As2—C33	1.949 (2)	C21—C22	1.397 (4)
C1—C2	1.391 (4)	C22—C23	1.396 (4)
C1—C6	1.393 (4)	C22—H22	0.9500
C2—C3	1.395 (4)	C23—C24	1.366 (5)
C2—H2	0.9500	C23—H23	0.9500
C3—C4	1.386 (5)	C24—C25	1.387 (4)
C3—H3	0.9500	C24—H24	0.9500
C4—C5	1.374 (5)	C25—C26	1.396 (4)
C4—H4	0.9500	C25—H25	0.9500
C5—C6	1.391 (4)	C26—H26	0.9500
C5—H5	0.9500	C27—C28	1.385 (3)
C6—H6	0.9500	C27—C32	1.396 (4)
C7—C12	1.384 (3)	C28—C29	1.395 (4)
C7—C8	1.398 (4)	C28—H28	0.9500
C8—C9	1.388 (4)	C29—C30	1.356 (5)
C8—H8	0.9500	C29—H29	0.9500
C9—C10	1.395 (5)	C30—C31	1.377 (5)
C9—H9	0.9500	C30—H30	0.9500
C10—C11	1.384 (5)	C31—C32	1.380 (4)
C10—H10	0.9500	C31—H31	0.9500
C11—C12	1.391 (4)	C32—H32	0.9500
C11—H11	0.9500	C33—C34	1.389 (4)
C12—H12	0.9500	C33—C38	1.399 (3)
C13—C18	1.383 (4)	C34—C35	1.393 (4)
C13—C14	1.403 (3)	C34—H34	0.9500
C14—C15	1.388 (4)	C35—C36	1.380 (4)
C14—H14	0.9500	C35—H35	0.9500
C15—C16	1.375 (4)	C36—C37	1.385 (5)
C15—H15	0.9500	C36—H36	0.9500
C16—C17	1.378 (4)	C37—C38	1.383 (4)
C16—H16	0.9500	C37—H37	0.9500
C17—C18	1.403 (4)	C38—H38	0.9500
			
C20—Pt1—C19	84.17 (12)	C13—C18—C17	120.2 (3)
C20—Pt1—As1	91.34 (8)	C13—C18—H18	119.9
C19—Pt1—As1	175.17 (8)	C17—C18—H18	119.9
C20—Pt1—As2	172.47 (8)	Pt1—C19—H19A	109.5
C19—Pt1—As2	88.30 (9)	Pt1—C19—H19B	109.5
As1—Pt1—As2	96.184 (10)	H19A—C19—H19B	109.5
C1—As1—C7	106.44 (10)	Pt1—C19—H19C	109.5
C1—As1—C13	100.90 (10)	H19A—C19—H19C	109.5
C7—As1—C13	99.16 (10)	H19B—C19—H19C	109.5
C1—As1—Pt1	114.16 (8)	Pt1—C20—H20A	109.5
C7—As1—Pt1	114.10 (7)	Pt1—C20—H20B	109.5
C13—As1—Pt1	120.02 (7)	H20A—C20—H20B	109.5
C27—As2—C21	103.04 (11)	Pt1—C20—H20C	109.5
C27—As2—C33	101.73 (11)	H20A—C20—H20C	109.5
C21—As2—C33	99.24 (11)	H20B—C20—H20C	109.5
C27—As2—Pt1	113.51 (8)	C26—C21—C22	119.6 (2)
C21—As2—Pt1	112.60 (8)	C26—C21—As2	122.77 (19)
C33—As2—Pt1	123.96 (7)	C22—C21—As2	117.67 (19)
C2—C1—C6	119.8 (2)	C23—C22—C21	119.4 (3)
C2—C1—As1	125.07 (19)	C23—C22—H22	120.3
C6—C1—As1	115.17 (19)	C21—C22—H22	120.3
C1—C2—C3	119.9 (3)	C24—C23—C22	120.8 (3)
C1—C2—H2	120.1	C24—C23—H23	119.6
C3—C2—H2	120.1	C22—C23—H23	119.6
C4—C3—C2	120.0 (3)	C23—C24—C25	120.2 (3)
C4—C3—H3	120.0	C23—C24—H24	119.9
C2—C3—H3	120.0	C25—C24—H24	119.9
C5—C4—C3	120.1 (3)	C24—C25—C26	119.7 (3)
C5—C4—H4	120.0	C24—C25—H25	120.1
C3—C4—H4	120.0	C26—C25—H25	120.1
C4—C5—C6	120.6 (3)	C21—C26—C25	120.3 (3)
C4—C5—H5	119.7	C21—C26—H26	119.9
C6—C5—H5	119.7	C25—C26—H26	119.9
C5—C6—C1	119.6 (3)	C28—C27—C32	118.8 (3)
C5—C6—H6	120.2	C28—C27—As2	117.8 (2)
C1—C6—H6	120.2	C32—C27—As2	123.44 (19)
C12—C7—C8	119.8 (2)	C27—C28—C29	119.9 (3)
C12—C7—As1	122.02 (19)	C27—C28—H28	120.0
C8—C7—As1	118.05 (19)	C29—C28—H28	120.0
C9—C8—C7	120.0 (3)	C30—C29—C28	120.8 (3)
C9—C8—H8	120.0	C30—C29—H29	119.6
C7—C8—H8	120.0	C28—C29—H29	119.6
C8—C9—C10	119.8 (3)	C29—C30—C31	119.9 (3)
C8—C9—H9	120.1	C29—C30—H30	120.1
C10—C9—H9	120.1	C31—C30—H30	120.1
C11—C10—C9	120.0 (3)	C30—C31—C32	120.5 (3)
C11—C10—H10	120.0	C30—C31—H31	119.8
C9—C10—H10	120.0	C32—C31—H31	119.8
C10—C11—C12	120.2 (3)	C31—C32—C27	120.2 (3)
C10—C11—H11	119.9	C31—C32—H32	119.9
C12—C11—H11	119.9	C27—C32—H32	119.9
C7—C12—C11	120.1 (3)	C34—C33—C38	118.6 (2)
C7—C12—H12	120.0	C34—C33—As2	121.24 (19)
C11—C12—H12	120.0	C38—C33—As2	120.0 (2)
C18—C13—C14	119.1 (2)	C33—C34—C35	120.4 (3)
C18—C13—As1	122.79 (19)	C33—C34—H34	119.8
C14—C13—As1	118.08 (19)	C35—C34—H34	119.8
C15—C14—C13	120.1 (3)	C36—C35—C34	120.2 (3)
C15—C14—H14	119.9	C36—C35—H35	119.9
C13—C14—H14	119.9	C34—C35—H35	119.9
C16—C15—C14	120.3 (3)	C35—C36—C37	120.0 (3)
C16—C15—H15	119.9	C35—C36—H36	120.0
C14—C15—H15	119.9	C37—C36—H36	120.0
C15—C16—C17	120.4 (3)	C38—C37—C36	119.9 (3)
C15—C16—H16	119.8	C38—C37—H37	120.0
C17—C16—H16	119.8	C36—C37—H37	120.0
C16—C17—C18	119.9 (3)	C37—C38—C33	120.8 (3)
C16—C17—H17	120.1	C37—C38—H38	119.6
C18—C17—H17	120.1	C33—C38—H38	119.6
